# Link between the referring physician and breast and cervical cancers screening: a cross-sectional study in France

**DOI:** 10.1186/s12875-023-02122-5

**Published:** 2023-08-29

**Authors:** Lisa Ouanhnon, Marie-Eve Rouge Bugat, Vladimir Druel, Pascale Grosclaude, Cyrille Delpierre

**Affiliations:** 1 Département Universitaire de Médecine Générale, Université Toulouse 3 Paul Sabatier, 37 allées Jules Guesde, 31000 Toulouse, France; 2 Equity Team : Labelled By the French League Against Cancer, UMR 1295 CERPOP, Inserm, Université Toulouse III, Toulouse, France; 3 Institut Claudius Regaud, IUCT-O, Registre Des Cancers du Tarn, F-31059 Toulouse, France

**Keywords:** General practitioners, Continuity of patient care, Early detection of cancer, Uterine cervical neoplasms, Breast neoplasms, Socioeconomic factors

## Abstract

**Background:**

The aims of the “médecin traitant” or referring physician (RP) reform, introduced in France in 2004, were to improve the organisation and quality of care and to allow for greater equity, particularly in terms of prevention**.** The objective of our study was to evaluate the effect of having a declared RP on the uptake of screening for breast and cervical cancers, and to explore the mechanisms involved.

**Methods:**

We used an existing dataset of 1,072,289 women, which combines data from the Health Insurance information systems, with census data. We built multivariable logistic regression models to study the effect of having a RP on the uptake of mammography and pap smear, adjusted for age, socio-economic level, health status and healthcare provision. We secondarily added to this model the variable “having consulted a General Practitioner (GP) within the year”. Finally, we evaluated the interaction between the effect of having a referring physician and the area of residence (metropolitan/urban/rural).

**Results:**

Patients who had a declared RP had a significantly higher uptake of mammography and pap smear than those who did not. The strength of the association was particularly important in very urban areas. The effect of having visited a GP seemed to explain a part of the correlation between having a RP and uptake of screening.

**Conclusions:**

Lower rates of gynaecological screening among women without an RP compared to those with an RP may partly reflect a specific behaviour pattern in women less adherent to the health care system. However, this result also shows the importance of the RP, who assumes the key role of relaying public health information in a more personalised and adapted way.

**Supplementary Information:**

The online version contains supplementary material available at 10.1186/s12875-023-02122-5.

## Background

The “médecin traitant” or referring physician (RP) reform was implemented in France in 2004. All individuals above 16 years old are encouraged to formally designate a RP to social insurance. The RP is responsible for coordinating the patient’s healthcare pathway. This gatekeeper-type model, inspired by practices in the United Kingdom [1], aimed to better control care pathways and health expenditure, improve healthcare quality and promote equity in healthcare [2]. In the last health and social insurance survey of the French Institute for Research and Documentation in Health Economics (IRDES), undertaken in 2014, around 96% of people had designated a RP [3].

A large majority of RPs in France are General Practitioners (GPs). GPs are in the best position to meet public health goals at an individual level [4]. They are supposed to know their patients in all their dimensions (biomedical, socio-cultural, psychological) and to take care of them in a global approach. They are the only physicians who can follow their patients on a regular basis at all stages of life. Furthermore, the financial and physical accessibility of GPs aims to reduce social and territorial inequalities, and therefore improve equity of care [5, 6].

In women’s health, one goal of the RP reform was to increase the uptake of breast and cervical cancer screening [7, 8]. In France, a nationally organised screening programme invites all women between 50 and 74 to have a mammography every 2 years [9]. For cervical cancer, pre-2018 guidelines recommended a pap smear every 3 years for women between 25 and 65. A national screening programme is progressively being implemented [10]. The participation rate is around 50% for breast cancer screening and 60% for cervical cancer [11]. The RPs, through their unique position as trusted healthcare professionals, are the first relay of key public health messages, particularly in the area of cancer prevention [12]. They can also carry out smear tests themselves.

The objective of our study was to evaluate the effect of having a RP on the uptake of breast and cervical cancers screening. We explored one of the potential mechanisms involved, the consultation of a GP within the year. Finally, we studied the territorial disparities in the RP effect on mammography and pap smear uptake.

## Methods

### Study design and population

**Fig. 1:**
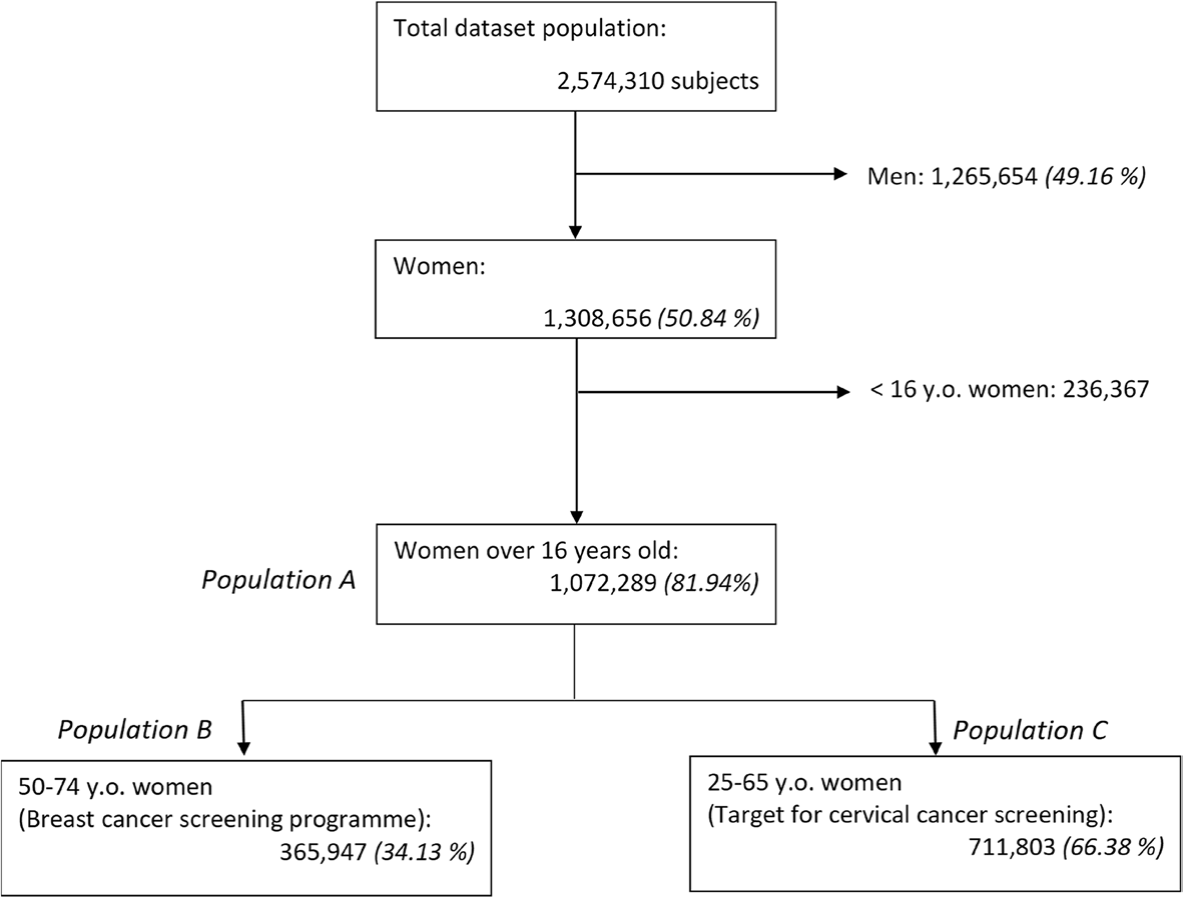
Flow chart. Identification of study populations **A**, **B** and **C**

We used a dataset combining prospectively collected health insurance data with census data [13]. This dataset included individuals who were beneficiaries of any of the three French health insurance providers on the 31^st^ of December 2012 in Midi-Pyrénées. The individuals with an incomplete address or with differences in the management of their data were excluded (which represents less than 2% of the population) [13]. We obtained a base of 2,574,310 subjects (88% of the region’s population). For this study, we focused on women over 16 years old, the age at which they are encouraged to formally designate a RP, and those targeted by breast or cervical cancer screening. Figure 1 shows the flow diagram with the different study populations.

### Variables and conceptual model

The variables chosen, as well as the methodology were described in a previously published article [13].

#### Main outcomes

Our outcome was, for each woman, the uptake of pap smear and mammography during the year, categorised as a binary variable for each screening test.

#### Main explanatory variable

We used a binary variable that discriminates between patients who had a designated RP and the ones who did not.

#### Covariates

##### Potential confounders

Patient’s age, categorised into 5-year groups, was considered as a potential confounder. As a proxy of the health status, we used ALD (“Affection de Longue Durée” or long-term condition), which is a co-payment exemption for patients suffering from a long-term condition. In the absence of individual social data, the socio-economic position (SEP) of the participants was approximated by an ecological deprivation index based on the person’s address, the European Deprivation Index (EDI) [14]. We used an EDI presentation in deciles: decile 1 corresponded to the least deprived zones in France, decile 10 to the most deprived. Healthcare accessibility to the two screening tests was another potential confounding factor. For mammography, we used the continuous variable of the distance to the nearest radiologist, which we categorised into terciles. For pap smear, we used the Potential Localised Accessibility (PLA) to the gynaecologist, a variable which is interpreted as gynaecologist density (number of full-time equivalents for 100 000 inhabitants) [15]. This continuous variable was categorised into terciles. For the descriptive analysis, we were also interested in the provision of GP care, approximated by the PLA to the GP.

##### Potentil mediating or moderating factors

Healthcare provision, transport facilities and type of medical practice are very different in rural and urban areas [16–18]. We assumed that the level of urbanisation of the place of residence could modify the impact of the RP on screening uptake. We built a variable to distinguish between Toulouse metropolitan area (the regional capital which covers almost a quarter of the region’s population), the other large urban areas (providing more than 10,000 jobs) and their suburbs, and the rest of the region [19]. Finally, to explore the link between RP and screening, we tried to disentangle the effect attributable to GP visits from the global effect of having a designated RP: we used a binary variable differentiating between women who had seen a GP at least once within the year and those who had not.

**Fig. 2:**
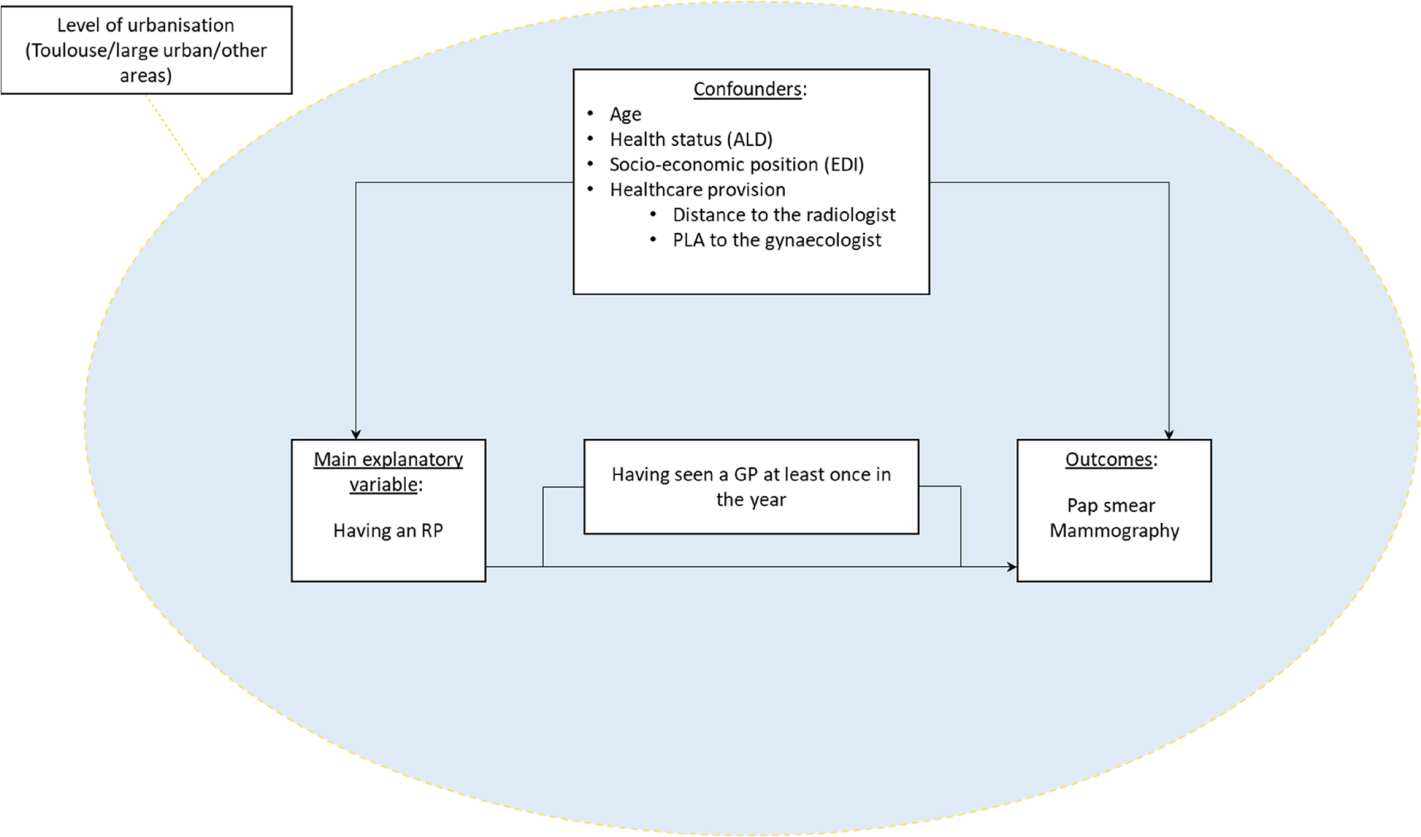
Conceptual model: Links between the studied variables assumed to explain the impact of “having an RP” on the uptake of screening, depending on the level of urbanisation

Our conceptual model showing how these variables are related is presented in Fig. 2.

### Statistical analysis

We conducted a descriptive analysis of the database. To better characterise the individuals without a RP, we performed univariate and multivariable analyses between covariates and “having a RP”. We tested the association between the main explanatory variable and the outcomes, and between each potential confounder and the outcomes. Secondly, we used a multivariable logistic regression model to analyse the association between having a RP and mammography and pap smear uptake, adjusted for all the previously identified confounders. We studied the interaction between having a RP and the type of place of residence in this model, and performed a subgroup analysis according to place of residence.

To disentangle the effect attributable to seeing a GP from the global effect of having an RP, we repeated the multivariable models (fully adjusted and stratified according to the place of residence), adding the variable "having seen a GP within the year".

Since we used data that are systematically recorded by health insurance providers, we expected very little missing data. This was therefore negligible compared to the global sample size (around 0.01%) and a complete case analysis was used.

Statistical analyses were performed with R software (R × 64 3.0.2) [20].

## Results

### Description of the population

Our study population consisted of 1,072,289 women over 16 years old, 112,593 women involved in the breast cancer screening programme and 205,072 in the cervical cancer screening programme. Our study population is described in Table 1 (population B and C) and Additional file 1: Table S1 (population A according to the place of residence). Among 50–74 women, 31% had a mammography within the year. Among women aged 25 to 65, 29% had a pap smear within the year. In the overall population, 91% of women had a designated RP.

**Table 1:** Characteristics of women targeted for breast and cervical cancer screening in Midi Pyrénées (2012). In population B: 50–74 women and population C: 25–65 women

	**Total** **50–74 y.o** ***N* = 365 947** **N (%)**	**No mammography** n (%) *n* = 253 354 (69.23)	** ≥ 1 mammography** n (%) *n* = 112 593 (30.77)	***p*-value**	**Total** **25–65 y.o** ***N* = 711 803** **N (%)**	**No pap smear** n (%) n = 506 731 (71.19)	** ≥ 1 pap smear** n (%) n = 205 072 (28.81%)	***p*-value**
**RP**^**a**^				*				*
No	20,032 (5.47)	18,963 (94.66)	1,069 (5.34)		57,596 (8.09)	52,948 (91.93)	4,648 (8.07)	
Yes	345,915 (94.53)	234,391 (67.76)	111,524 (32.24)		654,207 (91.91)	453,783 (69.36)	200,424 (30.64)	
**Age (/5 years)**				*				*
25–30 y.o	-	-	-		82,413 (11.58)	56,617 (68.7)	25,796 (31.3)	
30–35 y.o	-	-	-		88,249 (12.4)	58,932 (66.78)	29,317 (33.22)	
35–40 y.o	-	-	-		85,200 (11.97)	57,150 (67.08)	28,050 (32.92)	
40–45 y.o	-	-	-		92,964 (13.06)	63,042 (67.81)	29,922 (32.19)	
45–50 y.o	-	-	-		94,291 (13.25)	64,872 (68.8)	29,419 (31.2)	
50–55 y.o	88,241 (24.11)	61,449 (69.64)	26,792 (30.36)		88,241 (12.4)	64,145 (72.69)	24,096 (27.31)	
55–60 y.o	83,126 (22.72)	57,836 (69.58)	25,290 (30.42)		83,126 (11.68)	64,120 (77.14)	19,006 (22.86)	
60–65 y.o	81,209 (22.19)	55,168 (67.93)	26,041 (32.07)		81,209 (11.41)	64,544 (79.48)	16,665 (20.52)	
65–70 y.o	64,794 (17.71)	44,289 (68.35)	20,505 (31.65)		16,110 (2.26)^b^	13,309 (82.61)	2,801 (17.39)	
70–75 y.o	48,577 (13.27)	34,612 (71.25)	13,965 (28.75)		-	-	-	
**EDI**				*				*
1 (best)	31,201 (8.53)	20,675 (66.26)	10,526 (33.74)		62,238 (8.74)	40,787 (65.53)	21,451 (34.47)	
2	34,826 (9.52)	23,263 (66.8)	11,563 (33.2)		70,952 (9.97)	47,640 (67.14)	23,312 (32.86)	
3	30,111 (8.23)	20,414 (67.8)	9,697 (32.2)		60,763 (8.54)	41,703 (68.63)	19,060 (31.37)	
4	31,564 (8.63)	21,596 (68.42)	9,968 (31.58)		60,572 (8.51)	42,269 (69.78)	18,303 (30.22)	
5	32,733 (8.94)	22,750 (69.5)	9,983 (30.5)		65,031 (9.14)	46,072 (70.85)	18,959 (29.15)	
6	39,518 (10.8)	27,130 (68.65)	12,388 (31.35)		73,464 (10.32)	53,153 (72.35)	20,311 (27.65)	
7	38,825 (10.61)	27,107 (69.82)	11,718 (30.18)		72,276 (10.15)	52,119 (72.11)	20,157 (27.89)	
8	37,868 (10.35)	26,309 (69.48)	11,559 (30.52)		70,412 (9.89)	51,084 (72.55)	19,328 (27.45)	
9	42,390 (11.58)	29,998 (70.77)	12,392 (29.23)		82,232 (11.55)	60,646 (73.75)	21,586 (26.25)	
10 (worst)	46,911 (12.82)	34,112 (72.72)	12,799 (27.28)		93,863 (13.19)	71,258 (75.92)	22,605 (24.08)	
**GP PLA**				*				*
1 (worst)	11,427 (3.12)	8,212 (71.86)	3,215 (28.14)		18,607 (2.61)	13,784 (74.08)	4,823 (25.92)	
2	13,767 (3.76)	9,738 (70.73)	4,029 (29.27)		24,385 (3.43)	17,816 (73.06)	6,569 (26.94)	
3	14,455 (3.95)	10,195 (70.53)	4,260 (29.47)		26,121 (3.67)	18,888 (72.31)	7,233 (27.69)	
4	20,582 (5.62)	14,258 (69.27)	6,324 (30.73)		37,307 (5.24)	26,610 (71.33)	10,697 (28.67)	
5	26,405 (7.22)	18,029 (68.28)	8,376 (31.72)		49,815 (7)	35,139 (70.54)	14,676 (29.46)	
6	32,262 (8.82)	21,930 (67.97)	10,332 (32.03)		63,615 (8.94)	44,311 (69.65)	19,304 (30.35)	
7	50,863 (13.9)	34,371 (67.58)	16,492 (32.42)		98,949 (13.9)	68,782 (69.51)	30,167 (30.49)	
8	62,331 (17.03)	42,592 (68.33)	19,739 (31.67)		123,460 (17.34)	86,465 (70.03)	36,995 (29.97)	
9	64,131 (17.52)	44,615 (69.57)	19,516 (30.43)		127,253 (17.88)	90,793 (71.35)	36,460 (28.65)	
10 (best)	69,724 (19.05)	49,414 (70.87)	20,310 (29.13)		142,291 (19.99)	104,143 (73.19)	38,148 (26.81)	
**Urbanisation**				*				*
Toulouse Metropole	72,919 (19.93)	49,978 (68.54)	22,941 (31.46)		180,030 (25.59)	123,038 (68.34)	56,992 (31.66)	
Large urban areas	150,755 (41.2)	102,663 (68.1)	48,092 (31.9)		302,563 (42.51)	211,072 (69.76)	91,491 (30.24)	
Other area	142,273 (38.88)	100,713 (70.79)	41,560 (29.21)		229,210 (32.2)	172,621 (75.31)	56,589 (24.69)	
**GP consultation**				*				
No	62,262 (17.01)	53,043 (85.19)	9,219 (14.81)		154,322 (21.68)	130,475 (84.55)	23,847 (15.45)	
≥ 1 in the year	303,685 (82.99)	200,311 (65.96)	103,374 (34.04)		557,481 (78.32)	376,256 (67.49)	181,225 (32.51)	

*P<0.001

^a^*RP* Official referring physician

^b^Only 65 years women

### Association between covariates and RP

**Fig. 3:**
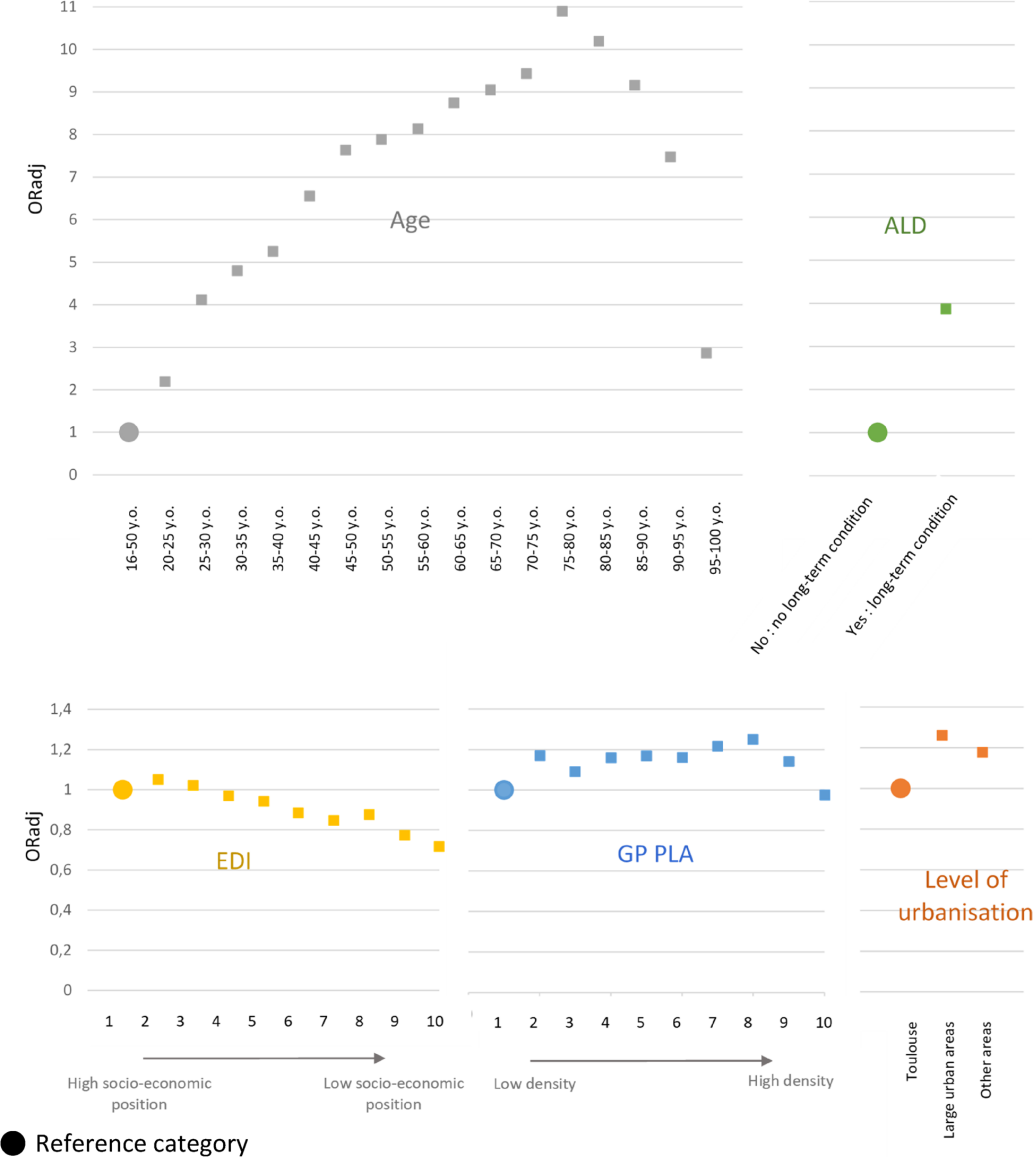
Determinants influencing the designation of a Referring Physician in Midi Pyrénées (2012): multivariable logistic regression model. Adjusted on age, long-term condition (ALD), EDI, GP PLA and level of urbanisation In population A: women over 16 years old (*n* = 1,072,289)

From the results of the univariate and multivariable analyses presented in Fig. 3 and Additional file 1: Table S2, we identified the profile of women without a designated RP. Younger women were less likely to have an RP, as well as healthier women (without ALD). A social gradient in the declaration of an RP was found: the likelihood of having an RP decreased with more disadvantaged socio-economic status (OR EDI 10 versus 1 = 0.72 95%CI [0.69; 0.74]). The proportion of women with an RP did not seem influenced by the accessibility to a GP but varied according to the area of residence: women living in Toulouse were less likely to have an RP than women living in the rest of the region (OR large urban areas vs Toulouse = 1.26 95%CI [1.24; 1.28]).

### Association between RP, covariates and screening

#### Univariate analyses

In the univariate analysis (Table 1 and Additional file 1: Table S3 and S4), we observed a strong association between having an RP and screening uptake (OR = 1.25, 8.44, 6.50 for mammography; OR = 5.62, 4.97, 4.68 for pap smear in Toulouse, other large urban areas and other areas respectively). The likelihood of screening uptake decreased with more disadvantaged socio-economic status (EDI 10 vs1 OR = 0.67, 0.77, 0.90 for mammography and OR = 0.57, 0.61, 0.72 for pap smear Toulouse, other large urban areas and other areas respectively). Regarding age, the mammography rate did not vary much throughout the recommended ages. Pap smear uptake decreased a lot after 55 (55–60 vs 20–25-year-old OR = 0.75, 0.66, 0.63 in Toulouse, other large urban areas and other areas respectively). We observed a lower rate of mammography and pap smear uptake in the most rural areas.

#### Multivariable analyses

Accounting for an interaction of area of residence on the relationship between having an RP and screening uptake improved our model statistically significantly (*p*-value = 2.2.10–16 for mammography and 1.9.10–4 for pap smear). We then stratified our analyses according to area of residence.

Table 2 presents the results of multivariable logistic regressions for mammography uptake, after stratification by area of residence. Having an RP had a major impact on the uptake of mammography, after adjustment for confounders. This result was found in all residential areas, with a particularly strong impact in Toulouse (OR = 14.094 95%CI [12.11; 16.524] in Toulouse, 8.387 95%CI [7.601; 9.282] in other large urban areas, and 6.376 95%CI [5.819; 7.003] elsewhere). The strength of this association remained unchanged after adjustment for confounders (OR = 14.25, 8.44, 6.50 before to 14.09, 8.40, 6.38 after adjustment in Toulouse, other large urban areas and other areas respectively).

**Table 2:** Mammography uptake according to the place of residence in Midi Pyrénées (2012): multivariable logistic regression model. Adjusted on designated RP, age, ALD (long-term condition), EDI, and distance to the radiologist. In population B: 50–74 women (*n* = 365,947)

		**Toulouse**		**Other large urban areas**		**Other areas**	
		Tot = 72,919	OR_adj_ (95%CI)	Tot = 150,755	OR_adj_ (95%CI)	Tot = 142,273	OR_adj_ (95%CI)
**Designated RP**	No^a^	4,898	1	7,428	1	7,706	1
	Yes	68,021	14.094 (12.11;16.524)	143,327	8.387 (7.601;9.282)	13,4567	6.376 (5.819;7.003)
**Age**	50–55 y.o.^a^	19,112	1	37,568	1	31,561	1
	55–60 y.o	17,097	0.934 (0.893;0.977)	34,985	1.004 (0.973;1.036)	31,044	1.039 (1.003;1.076)
	60–65 y.o	15,774	0.987 (0.942;1.033)	33,460	1.078 (1.045;1.113)	31,975	1.123 (1.085;1.163)
	65–70 y.o	12,305	0.982 (0.935;1.032)	25,825	1.047 (1.011;1.083)	26,664	1.092 (1.053;1.133)
	70–74 y.o	8,631	0.878 (0.829;0.929)	18,917	0.881 (0.847;0.915)	21,029	0.962 (0.924;1.001)
**ALD** (long-term condition)	No^a^	57,407	1	117,379	1	110,515	1
	Yes	15,512	1.028 (0.988;1.069)	33,376	1.034 (1.007;1.062)	31,758	1.065 (1.036;1.095)
**EDI** (deciles)	1^a^	7,886	1	20,596	1	2,719	1
	2	8,615	0.959 (0.898;1.023)	20,315	0.983 (0.943;1.024)	5,896	1.064 (0.963;1.177)
	3	4,436	0.922 (0.852;0.997)	15,563	0.982 (0.939;1.026)	10,112	1.05 (0.956;1.153)
	4	3,484	0.864 (0.793;0.941)	14,848	0.956 (0.913;1.001)	13,232	1.062 (0.97;1.165)
	5	8,183	0.823 (0.77;0.879)	11,820	0.916 (0.872;0.962)	12,730	1.002 (0.914;1.1)
	6	3,368	0.869 (0.796;0.948)	16,244	0.951 (0.91;0.994)	19,906	1.075 (0.984;1.176)
	7	6,678	0.864 (0.804;0.928)	12,055	0.846 (0.805;0.889)	20,092	1.062 (0.972;1.161)
	8	6,367	0.822 (0.763;0.886)	10,760	0.885 (0.841;0.932)	20,741	1.047 (0.959;1.145)
	9	9,519	0.742 (0.693;0.793)	12,192	0.901 (0.856;0.948)	20,679	0.967 (0.885;1.057)
	10	14,383	0.673 (0.634;0.715)	16,362	0.771 (0.734;0.81)	16,166	0.886 (0.809;0.97)
**Distance (time) to the radiologist** (terciles)	1^a^	24,324	1	50,709	1	47,439	1
	2	24,510	0.986 (0.947;1.027)	50,400	1.03 (0.999;1.061)	47,456	0.884 (0.859;0.909)
	3	24,085	0.938 (0.899;0.979)	49,646	0.956 (0.928;0.985)	47,378	0.872 (0.847;0.897)

^a^Reference Category

Table 3 presents the results of multivariable logistic regressions for pap smear uptake, after stratification by area of residence. A major impact of having an RP on the uptake of pap smear was also observed after adjustment for confounders. This effect was found in all areas of residence (OR = 5.838 95%CI [5.54; 6.156] in Toulouse, 5.254 95%CI [5.00; 5.52] in other large urban areas and 5.099 95%CI [4.814; 5.406] elsewhere). The strength of this association changed minimally after adjustment (OR 5.62, 4.97, 4.68 before to 5.84, 5.25, 5.10 after adjustment in Toulouse, other large urban areas and other areas respectively).

**Table 3:** Pap smear uptake according to the place of residence in Midi Pyrénées (2012): multivariable logistic regression model, Adjusted on designated RP, age, ALD (long-term condition), EDI, and PLA to the gynaecologist. In population C: 25–65 women (n = 711,803)

		**Toulouse**		**Other large urban areas**		**Other areas**	
		Tot = 180,030	OR_adj_ (95%CI)	Tot = 302,563	OR_adj_ (95%CI)	Tot = 229,210	OR_adj_ (95%CI)
**Designated RP**	No^a^	18,754	1	20,659	1	18,183	1
	Yes	161,276	5.838 (5.54;6.156)	281,904	5.254 (5.002;5.523)	211,027	5.099 (4.814;5.406)
**Age**	25–30 y.o.^a^	30,798	1	32,111	1	19,504	1
	30–35 y.o	28,146	1.04 (1.004;1.077)	36,721	1.092 (1.058;1.128)	23,382	1.064 (1.019;1.11)
	35–40 y.o	23,292	0.996 (0.959;1.033)	37,351	1.075 (1.041;1.11)	24,557	1.032 (0.989;1.076)
	40–45 y.o	21,537	0.973 (0.937;1.011)	41,983	1.025 (0.994;1.058)	29,444	0.955 (0.917;0.995)
	45–50 y.o	21,259	0.982 (0.946;1.02)	41,829	0.959 (0.929;0.99)	31,203	0.885 (0.85;0.922)
	50–55 y.o	19,112	0.842 (0.809;0.876)	37,568	0.785 (0.76;0.811)	31,561	0.747 (0.716;0.778)
	55–60 y.o	17,097	0.673 (0.645;0.702)	34,985	0.622 (0.601;0.644)	31,044	0.59 (0.566;0.616)
	60–65 y.o	15,774	0.595 (0.569;0.622)	33,460	0.538 (0.519;0.558)	31,975	0.52 (0.498;0.543)
	65 y.o	3,015	0.537 (0.49;0.587)	6,555	0.43 (0.401;0.46)	6,540	0.416 (0.385;0.449)
**ALD** (long-term condition)	No^a^	162,439	1	26,8275	1	201,462	1
	Yes	17,591	0.835 (0.806;0.866)	34,288	0.86 (0.838;0.883)	27,748	0.863 (0.836;0.891)
**EDI** (deciles)	1^a^	14,747	1	42,750	1	4,741	1
	2	19,389	0.855 (0.817;0.894)	41,657	0.958 (0.93;0.986)	9,906	0.971 (0.898;1.05)
	3	10,922	0.846 (0.802;0.892)	32,952	0.918 (0.89;0.947)	16,889	0.95 (0.883;1.022)
	4	8,239	0.837 (0.79;0.886)	30,690	0.91 (0.881;0.939)	21,643	0.924 (0.86;0.992)
	5	21,020	0.777 (0.742;0.813)	23,450	0.812 (0.784;0.841)	20,561	0.894 (0.832;0.961)
	6	9,173	0.762 (0.719;0.807)	32,475	0.814 (0.789;0.84)	31,816	0.879 (0.82;0.942)
	7	17,062	0.802 (0.762;0.843)	23,586	0.788 (0.761;0.816)	31,628	0.876 (0.818;0.939)
	8	17,051	0.787 (0.749;0.828)	20,967	0.795 (0.766;0.825)	32,394	0.85 (0.793;0.911)
	9	26,337	0.73 (0.695;0.766)	22,732	0.721 (0.695;0.748)	33,163	0.813 (0.759;0.872)
	10	36,090	0.59 (0.563;0.618)	31,304	0.653 (0.631;0.676)	26,469	0.734 (0.684;0.788)
**PLA to the gynaecologist** (terciles)	1^a^	60,220	1	101,380	1	76,583	1
	2	60,824	1.045 (1.016;1.075)	102,717	1.073 (1.052;1.094)	78,139	1.082 (1.057;1.108)
	3	58,986	0.956 (0.926;0.987)	98,466	1.03 (1.009;1.051)	74,488	1.201 (1.173;1.231)

^a^Reference category

#### Models with the variable “GP consultation”

A large majority of patients with a declared RP had seen a GP within the year, unlike those without an RP (OR = 10.18 95%CI [10.04; 10.33], Additional file 1: Table S5). In addition, there was an association between GP consultation and the uptake of screening tests (≥ 1 GP consultation vs 0 OR = 2.97 95%CI [2.90; 3.04] for mammography; OR = 2.64 95%CI [2.60; 2.67] for pap smear, Additional file 1: Table S6 and S7). Additional file 1: Table S6 and S7 show that even if they consulted a GP, women without an RP seem to have a lower uptake of screenings than those with an RP (17.72% without RP vs 34.28% for mammography; 19.13% vs 32.91% for pap smear). Table 4 presents the results of stratified multivariable logistic regressions adjusted on the same confounders than previously, then adding the variable "GP consultation within the year". The GP visit was associated with a higher probability of undertaking both screenings in the fully adjusted models. The impact of having an RP on the uptake of screenings decreased when the variable "GP consultation within the year" was added, but remained important.

**Table 4:** Effect attributable to seeing a GP from the global effect of having an RP on screening uptake according to the place of residence in Midi Pyrénées (2012): multivariable logistic regression models with and without the variable “GP consultation within the year". Adjusted on the previously identified confounding factors (Table 2 for mammography and Table 3 for pap smear). In population B for mammography and C for pap-smear

**Mammography**							
		**Toulouse** OR_adj_ (95%CI)		**Large urban areas** OR_adj_ (95%CI)		**Other areas** OR_adj_ (95%CI)	
		*Adjusted on the same confounding factors as in *Table 2	*Adjusted on the same factors* + *GP consultation within the year*	*Adjusted on the same confounding factors as in *Table 2	*Adjusted on the same factors* + *GP consultation within the year*	*Adjusted on the same confounding factors as in *Table 2	*Adjusted on the same factors* + *GP consultation within the year*
**Designated RP**	No^a^ Yes	1 14.094 (12.11; 16.524)	1 8.156 (6.979; 9.596)	1 8.387 (7.601;9.282)	1 5.214 (4.714; 5.783)	1 6.376 (5.819;7.003)	1 3.951 (3.597; 4.349)
** ≥ 1 GP consultation** within the year	No^a^ Yes		1 2.32 (2.197; 2.45)		1 2.252 (2.169; 2.339)		1 2.485 (2.389; 2.586)
**Pap smear**							
		**Toulouse** OR_adj_ (95%CI)		**Large urban areas** OR_adj_ (95%CI)		**Other areas** OR_adj_ (95%CI)	
		*Adjusted on the same confounding factors as in *Table 3	*Adjusted on the same factors* + *GP consultation within the year*	*Adjusted on the same confounding factors as in *Table 3	*Adjusted on the same factors* + *GP consultation within the year*	*Adjusted on the same confounding factors as in *Table 3	*Adjusted on the same factors* + *GP consultation within the year*
**Designated RP**	No^a^ Yes	1 5.838 (5.54;6.156)	1 3.681 (3.485; 3.89)	1 5.254 (5.002;5.523)	1 3.653 (3.472; 3.845)	1 5.099 (4.814;5.406)	1 3.602 (3.396; 3.824)
** ≥ 1 GP consultation** within the year	No^a^ Yes		1 2.436 (2.364; 2.51)		1 2.171 (2.12; 2.224)		1 2.231 (2.168; 2.296)

^a^Reference category

## Discussion

In this study, we found that patients with a designated RP had a significantly higher uptake of breast and cervical cancers screening than those without. The strength of the association between having an RP and the mammography uptake varied according to the level of urbanisation of the area of residence, and was particularly important in Toulouse. This variation was less pronounced for pap smear. The effect of having an RP on screening uptake was only partially explained by having consulted a GP.

The main strength of our study is its power and comprehensiveness, achieved by using health insurance data. The use of EDI is also a strength: this variable has already proven its effectiveness in approximating the level of deprivation, that is usually difficult to capture in French healthcare databases [12]. Our study also has limitations. As our data covered only 1 year, we could not differentiate between women who had screening tests every year (more often than recommended) and the ones who had tests every two and three years as recommended. As our study is cross-sectional, we could not investigate true causal links. The database did not include which healthcare provider performed the pap smears. The effect of having an RPs performing pap smears themselves could not be evaluated, nor could the effect of the GP gender or age.

This study’s main result is the major impact of having an RP on the uptake of screening. Different mechanisms could explain this effect, including an increased use of GP consultation. This may correspond to both a direct effect of the GP performing the screening test (especially for the pap smear but also prescribing mammography), but also an indirect effect of the GP reminding, informing and promoting these tests to the patients. Primary care has already demonstrated its effectiveness in prevention [5, 21]. Our results suggest that the impact of the GP consultation could be increased if the doctor consulted is the RP. In fact, the RP often knows the patient well, is chosen by her and often has a strong bond with her. Several studies have looked specifically at the impact of continuity of care: patients who are followed up over time with the same GP show higher satisfaction, better compliance with care, prevention [22] and, specifically, in their screening tests uptake [23]. This impact is even more important if the RP is chosen by the patient and consulted regularly [22].

However, the effect of having an RP on the screening uptake does not seem fully explained by the GP consultation effect. This could be due to the different health behaviours of women who have an RP and those who do not: having an RP could be, in itself, a sign of good adherence to the healthcare system. This assumption raises the question of the characteristics of women who do not have an RP. In our study, we confirmed that the likelihood of having a designated RP decreased with age and socio-economic status [2]. Age (young or elderly people) [24–26] and low socio-economic level [27–29] are known to have poorer adherence to healthcare.

Our results also suggest that the strength of the association between having an RP and screening varies with the level of urbanisation of the patient’s area of residence. This association was particularly strong in Toulouse. The smaller impact of the RP in more rural areas can be explained by the lack of availability of GPs, who are in shorter supply in these places, forcing them to focus on acute care at the expense of prevention [1]. In addition, rural GPs are more likely to be male and older [30], and several studies have shown that patients treated by female and middle-aged doctors have a higher recourse to gynaecological screening [31, 32].

Our study suggests an association between deprivation and screening uptake, confirming the result of previous studies around the world [28, 33–37]. A social gradient was also found in the designation of an RP. To improve their uptake of cancers screening, the most disadvantaged women should have access to a structured and adapted care pathway, built around their RP. But for these populations, even GPs do not seem to have much impact on prevention. It is proven difficult for them to propose appropriate health education to this category of population [38].

This study suggests various ways to improve the uptake of gynaecological screening for all women, and in particular the most disadvantaged:

- Implementing actions to encourage women who do not have an RP to appoint one (letters, assistance in choosing an RP and support).
- Supporting continuity of care: regular consultations with the same GP would allow better access to prevention for all.
- Improving the remuneration of consultations dedicated to prevention by the national health insurance, to avoid that preventive care is provided only after a "curative" consultation [28].
- There are many barriers to health education and prevention by RP in low socio-economic populations [38]. Particular attention must be paid to them to avoid aggravating the already existing social inequalities in health. It is essential that RPs improve their communication skills, adapt to the literacy level of each patient, and take every opportunity to bring up prevention.

## Conclusion

Lower rates of gynaecological screening among women without an RP compared to those with an RP may partly reflect a specific behaviour pattern in women less adherent to the health care system. However, this study also shows the importance of continuity of care and of the RP, who assumes the key role of relaying public health information in a more personalised and adapted way.

Databases reflecting adherence to the health care system and more precise characteristics of RPs (gender, age, pap smears delivery, consultations dedicated to prevention) could enable us further analyses, by helping identify the RP's own role and the mechanisms to increase women’s uptake of screening.

### Supplementary Information

**Additional file 1.**

## Data Availability

The data we used in this study belongs to the French National Health Insurance. The procurement of such data necessitates the agreement of the French National Institute of Health Data (INDS) and the permission from the ‘Commission Nationale Informatique et Liberte´s’ (CNIL) which is the French Data Protection Authority in accordance with Law No 78/17 of 6 January 1978 on computing, files and personal information, article 54, paragraph I. Data cannot be diffused without these authorisations. A CNIL Authorisation (no. 1634837) was obtained for our study. In addition, data cannot be shared with anyone who does not have these authorisations. In our study, the Regional Health Agency of Occitanie completed the necessary formalities with the relevant authorities. If other authors want to obtain the data, they have to contact directly the French National Institute of Health Data and obtain the permission of the CNIL. It can be done on the INDS website (http://www.indsante.fr/). In addition, data regarding demographical characteristics of the whole inhabitants of the region can be freely obtained from the French national institute for statistics and economic studies (https://www.insee.fr/fr/statistiques/).

## Abbreviations

ALD: Affection de Longue Durée or long-term condition
CMU-C: Couverture Médicale Universelle-complémentaire (Universal supplementary coverage, in addition to basic national insurance coverage)
EDI: European Deprivation Index
GP: General Practitioner
IRDES: Institute for Research and Documentation in Health Economics
RP: Referring Physician
SEP: Socio-economic position
