# Supplementary material for: Link between the referring physician and breast and cervical cancers screening: a cross-sectional study in France
Source: BMC Prim Care. 2023 Aug 29;24:167. doi: 10.1186/s12875-023-02122-5 (PMC10464303; doi:10.1186/s12875-023-02122-5)
Supplement: Supplementary file 1 — Additional file 1. [file 12875_2023_2122_MOESM1_ESM.pdf]

Additional file 1

Table 1: **Characteristics of women over 16 years old in Midi Pyrénées, according to the place of residence**

In population A: women over 16 years old (n= 1,072,289), 2012

	**Total** **n (%)** n= 1,072,289	**Toulouse** **n (%)** n= 255,329 (23.81%)	**Large urban areas** **n (%)** n= 445,014 (41.50%)	**Other areas** **n (%)** n= 371,946 (34.69%)
**Mammography in the past year**				
0	905,277 (84.42)	218,420 (85.54)	371,933 (83.58)	314,924 (84.67)
≥1	167,012 (15.58)	36,909 (14.46)	73,081 (16.42)	57,022 (15.33)
among 50-74 y.o. (365,947)	*112,593 (30.77)*	*22,941 (31.46)*	*48,092 (31.90)*	*41,560 (29.21)*
**Pap smear in the past year**				
0	837,164 (78.07)	189,839 (74.35)	340,955 (76.62)	306,370 (82.37)
≥1	235,125 (21.93)	65,490 (25.65)	104,059 (23.38)	65,576 (17.63)
among 25-65 y.o. (711,803)	*205,072 (28.81)*	*56,992 (31.66)*	*91,491 (30.24)*	*56,589 (24.69)*
**Designated RP**				
No	98,258 (9.16)	30,199 (11.83)	36,798 (8.27)	31,261 (8.40)
Yes	974,031 (90.84)	225,130 (88.17)	408,216 (91.73)	340,685 (91.60)
**Age**				
16-20 y.o.	45,262 (4.22)	10,195 (3.99)	20,332 (4.57)	14,735 (3.96)
20-25 y.o.	63,068 (5.88)	20,599 (8.07)	25,719 (5.78)	16,750 (4.5)
25-30 y.o.	82,413 (7.69)	30,798 (12.06)	32,111 (7.22)	19,504 (5.24)
30-35 y.o.	88,249 (8.23)	28,146 (11.02)	36,721 (8.25)	23,382 (6.29)
35-40 y.o.	85,200 (7.95)	23,292 (9.12)	37,351 (8.39)	24,557 (6.60)
40-45 y.o.	92,964 (8.67)	21,537 (8.43)	41,983 (9.43)	29,444 (7.92)
45-50 y.o.	94,291 (8.79)	21,259 (8.33)	41,829 (9.40)	31,203 (8.39)
50-55 y.o.	88,241 (8.23)	19,112 (7.49)	37,568 (8.44)	31,561 (8.49)
55-60 y.o.	83,126 (7.75)	17,097 (6.70)	34,985 (7.86)	31,044 (8.35)
60-65 y.o.	81,209 (7.57)	15,774 (6.18)	33,460 (7.52)	31,975 (8.6)
65-70 y.o.	64,794 (6.04)	12,305 (4.82)	25,825 (5.80)	26,664 (7.17)
70-75 y.o.	48,577 (4.53)	8,631 (3.38)	18,917 (4.25)	21,029 (5.65)
75-80 y.o.	50,815 (4.74)	8,793 (3.44)	19,298 (4.34)	22,724 (6.11)
80-85 y.o.	48,148 (4.49)	8,049 (3.15)	18,163 (4.08)	21,936 (5.9)
85-90 y.o.	34,698 (3.24)	5,890 (2.31)	12,760 (2.87)	16,048 (4.31)
90-95 y.o.	16,602 (1.55)	2,927 (1.15)	6,229 (1.40)	7,446 (2.00)
95-100 y.o.	4,632 (0.43)	925 (0.36)	1,763 (0.40)	1,944 (0.52)
**EDI (deciles)**				
1 (best)	85,793 (8)	20,810 (8.15)	58,041 (13.04)	6,942 (1.87)
2	100,164 (9.34)	26,825 (10.51)	58,530 (13.15)	14,809 (3.98)
3	87,506 (8.16)	15,253 (5.97)	46,566 (10.46)	25,687 (6.91)
4	89,339 (8.33)	11,428 (4.48)	43,729 (9.83)	34,182 (9.19)
5	96,394 (8.99)	28,997 (11.36)	34,869 (7.84)	32,528 (8.75)
6	113,041 (10.54)	12,966 (5.08)	48,252 (10.84)	51,823 (13.93)
7	112,486 (10.49)	24,398 (9.56)	35,706 (8.02)	52,382 (14.08)
8	110,300 (10.29)	24,453 (9.58)	32,162 (7.23)	53,685 (14.43)
9	129,232 (12.05)	37,884 (14.84)	36,389 (8.18)	54,959 (14.78)
10 (worst)	148,034 (13.81)	52,315 (20.49)	50,770 (11.41)	44,949 (12.08)
**GP PLA (deciles)**				
1 (worst)	28,746 (2.68)	1,118 (0.44)	4,594 (1.03)	23,034 (6.19)
2	36,820 (3.43)	3,091 (1.21)	13,642 (3.07)	20,087 (5.4)
3	38,611 (3.60)	0 (0)	17,944 (4.03)	20,667 (5.56)
4	55,032 (5.13)	2,371 (0.93)	26,811 (6.02)	25,850 (6.95)
5	73,046 (6.81)	4,676 (1.83)	40,593 (9.12)	27,777 (7.47)
6	92,524 (8.63)	9,227 (3.61)	51,311 (11.53)	31,986 (8.60)
7	145,227 (13.54)	28,333 (11.1)	75,106 (16.88)	41,788 (11.23)
8	184,884 (17.24)	51,400 (20.13)	74,876 (16.83)	58,608 (15.76)
9	195,551 (18.24)	55,027 (21.55)	84,401 (18.97)	56,123 (15.09)
10 (best)	221,848 (20.69)	100,086 (39.20)	55,736 (12.52)	66,026 (17.75)
**ALD (long-term condition)**				
No	880,950 (82.16)	218,950 (85.75)	36,8132 (82.72)	293,868 (79.01)
Yes	191,339 (17.84)	36,379 (14.25)	76,882 (17.28)	78,078 (20.99)

Table 2: **Determinants influencing the designation of a Referring Physician in Midi Pyrénées (2012): univariate and multivariable logistic regression analyses**

Multivariable logistic regression adjusted on age, long-term condition (ALD), EDI, GP PLA and level of urbanisation

In population A: women over 16 years old (n= 1,072,289)

	**Total**	**Univariate analysis**		**Multivariable analysis**	
	**n (%)**	OR	95%CI	OR_{adj}	95%CI
**Age**					
16-20 y.o.[1]	45,262 (4.22)	1		1	
20-25 y.o.	63,068 (5.88)	2.056	(2.00; 2.11)	2.19	(2.13; 2.25)
25-30 y.o.	82,413 (7.69)	3.833	(3.73; 3.94)	4.11	(4.00; 4.23)
30-35 y.o.	88,249 (8.23)	4.642	(4.51; 4.78)	4.80	(4.66; 4.94)
35-40 y.o.	85,200 (7.95)	5.284	(5.13; 5.44)	5.25	(5.10; 5.41)
40-45 y.o.	92,964 (8.67)	6.82	(6.61; 7.03)	6.56	(6.36; 6.76)
45-50 y.o.	94,291 (8.79)	8.132	(7.88; 8.40)	7.64	(7.39; 7.89)
50-55 y.o.	88,241 (8.23)	8.612	(8.33; 8.90)	7.88	(7.63; 8.15)
55-60 y.o.	83,126 (7.75)	9.174	(8.86; 9.50)	8.14	(7.86; 8.43)
60-65 y.o.	81,209 (7.57)	10.206	(9.84; 10.58)	8.74	(8.43; 9.07)
65-70 y.o.	64,794 (6.04)	11.043	(10.61; 11.50)	9.05	(8.69; 9.43)
70-75 y.o.	48,577 (4.53)	12.203	(11.64; 12.79)	9.43	(8.99; 9.89)
75-80 y.o.	50,815 (4.74)	15.116	(14.38; 15.89)	10.90	(10.36; 11.47)
80-85 y.o.	48,148 (4.49)	15.351	(14.58; 16.17)	10.19	(9.67; 10.75)
85-90 y.o.	34,698 (3.24)	15.278	(14.40; 16.22)	9.16	(8.62; 9.74)
90-95 y.o.	16,602 (1.55)	13.178	(12.18; 14.26)	7.48	(6.91; 8.11)
95-100 y.o.	4,632 (0.43)	5.036	(4.57; 5.55)	2.86	(2.59; 3.17)
**EDI**					
1[1] (high SEP)	85,793 (8)	1		1	
2	100,164 (9.34)	1.026	(0.99;1.06)	1.05	(1.01; 1.09)
3	87,506 (8.16)	1.021	(0.99;1.06)	1.02	(0.98; 1.06)
4	89,339 (8.33)	0.988	(0.96;1.02)	0.97	(0.93; 1.01)
5	96,394 (8.99)	0.898	(0.87;0.93)	0.94	(0.91; 0.98)
6	113,041 (10.54)	0.919	(0.89;0.95)	0.88	(0.85; 0.92)
7	112,486 (10.49)	0.833	(0.81;0.86)	0.85	(0.82; 0.88)
8	110,300 (10.29)	0.849	(0.82;0.88)	0.88	(0.85; 0.91)
9	129,232 (12.05)	0.721	(0.70;0.74)	0.77	(0.75; 0.80)
10 (low SEP)	148,034 (13.81)	0.627	(0.61;0.65)	0.72	(0.69; 0.74)
**Level of urbanisation**					
Toulouse Métropole[1]	255,329 (23.81)	1		1	
Large urban areas	445,014 (41.50)	1.488	(1.46; 1.51)	1.26	(1.24; 1.28)
Other areas	371,946 (34.69)	1.462	(1.44; 1.49)	1.18	(1.16; 1.20)
**GP PLA**					
1[1] (low density)	28,746 (2.68)	1		1	
2	36,820 (3.43)	1.139	(1.08; 1.20)	1.17	(1.11; 1.24)
3	38,611 (3.60)	1.091	(1.03; 1.15)	1.09	(1.03; 1.15)
4	55,032 (5.13)	1.162	(1.11; 1.22)	1.16	(1.10; 1.22)
5	73,046 (6.81)	1.14	(1.09; 1.20)	1.17	(1.11; 1.23)
6	92,524 (8.63)	1.11	(1.06; 1.16)	1.16	(1.10; 1.22)
7	145,227 (13.54)	1.121	(1.07; 1.17)	1.22	(1.16; 1.27)
8	184,884 (17.24)	1.069	(1.02; 1.12)	1.25	(1.19; 1.31)
9	195,551 (18.24)	0.954	(0.91; 1.00)	1.14	(1.09; 1.19)
10 (high density)	221,848 (20.69)	0.737	(0.71; 0.77)	0.98	(0.93; 1.02)
**ALD** (long-term condition)					
No[1]	880,950 (82.16)	1		1	
Yes	191,339 (17.84)	5.893	(5.70; 6.09)	3.87	(3.74; 4.01)

[1]*: Reference Category*

Table 3: **Mammography uptake according to the place of residence in Midi Pyrénées (2012): univariate logistic regression model**

In population B: 50-74 women (n=365,947)

		**TOULOUSE** OR (95%CI)	**OTHER LARGE URBAN AREAS** OR (95%CI)	**OTHER AREAS** OR (95%CI)
**DESIGNATED RP**	No[1]	1	1	1
	Yes	14.25 (12.24; 16.70)	8.44 (7.65; 9.34)	6.50 (5.93; 7.13)
**AGE**	50-55 y.o.[1]	1	1	1
	55-60 y.o.	0.93 (0.89; 0.97)	1.01 (0.98; 1.04)	1.05 (1.01; 1.08)
	60-65 y.o.	1.00 (0.95; 1.04)	1.09 (1.06; 1.12)	1.14 (1.10; 1.18)
	65-70 y.o.	1.00 (0.95; 1.05)	1.06 (1.02; 1.10)	1.13 (1.09; 1.17)
	70-74 y.o.	0.89 (0.84; 0.94)	0.90 (0.86; 0.93)	1.00 (0.97; 1.04)
**ALD**	No[1]	1	1	1
	Yes	1.06 (1.02; 1.10)	1.07 (1.04; 1.10)	1.12 (1.09; 1.15)
**EDI** (DECILES)	1[1]	1	1	1
	2	0.97 (0.91; 1.03)	0.98 (0.94; 1.02)	1.07 (0.97; 1.19)
	3	0.93 (0.86; 1.00)	0.99 (0.94; 1.03)	1.04 (0.94; 1.14)
	4	0.88 (0.81; 0.96)	0.95 (0.91; 1.00)	1.06 (0.97; 1.16)
	5	0.83 (0.78; 0.89)	0.91 (0.87; 0.96)	1.04 (0.96; 1.14)
	6	0.87 (0.80; 0.95)	0.95 (0.91; 0.99)	1.06 (0.97; 1.16)
	7	0.86 (0.80; 0.92)	0.84 (0.80; 0.88)	1.04 (0.96; 1.14)
	8	0.83 (0.77; 0.89)	0.89 (0.84; 0.93)	1.05 (0.96; 1.15)
	9	0.75 (0.70; 0.80)	0.90 (0.85; 0.94)	0.96 (0.88; 1.05)
	10	0.67 (0.64; 0.71)	0.77 (0.73; 0.80)	0.90 (0.82; 0.98)
**DISTANCE (TIME) TO THE RADIOLOGIST** (TERCILES)	1[1]	1	1	1
	2	1.01 (0.97; 1.05)	1.12 (1.10; 1.15)	0.88 (0.86; 0.91)
	3	1.04 (1.00; 1.08)	1.02 (1.00; 1.05)	0.87 (0.84; 0.89)

[1]*: Reference Category*

Table 4: **Pap smear uptake according to the place of residence in Midi Pyrénées (2012): Univariate logistic regression model**

In population C: 25-65 women (n=711,803)

		**TOULOUSE** OR (95%CI)	**OTHER LARGE URBAN AREAS** OR (95%CI)	**OTHER AREAS** OR (95%CI)
**DESIGNATED RP**	No[1]	1	1	1
	Yes	5.62 (5.33; 5.92)	4.97 (4.73; 5.23)	4.68 (4.44; 4.98)
**AGE**	25-30 y.o.1	1	1	1
	30-35 y.o.	1.07 (1.04; 1.11)	1.13 (1.09; 1.16)	1.08 (1.04; 1.13)
	35-40 y.o.	1.05 (1.01; 1.09)	1.13 (1.09; 1.17)	1.06 (1.02; 1.10)
	40-45 y.o.	1.07 (1.03; 1.11)	1.09 (1.06; 1.13)	1.00 (0.96; 1.04)
	45-50 y.o.	1.10 (1.06; 1.14)	1.03 (1.00; 1.06)	0.93 (0.88; 0.97)
	50-55 y.o.	0.95 (0.91; 0.98)	0.84 (0.81; 0.86)	0.79 (0.75; 0.82)
	55-60 y.o.	0.75 (0.72; 0.79)	0.66 (0.64; 0.69)	0.63 (0.60; 0.65)
	60-65 y.o.	0.67 (0.64; 0.70)	0.57 (0.55; 0.59)	0.55 (0.53; 0.58)
	65 y.o.	0.61 (0.55; 0.66)	0.45 (0.42; 0.49)	0.44 (0.41; 0.47)
**ALD**	No[1]	1	1	1
	Yes	0.80 (0.77; 0.82)	0.77 (0.75; 0.79)	0.79 (0.77; 0.81)
**EDI** (DECILES)	1[1]	1	1	1
	2	0.88 (0.84; 0.93)	0.96 (0.94; 0.99)	0.97 (0.90; 1.05)
	3	0.88 (0.84; 0.93)	0.93 (0.90; 0.96)	0.95 (0.88; 1.02)
	4	0.86 (0.81; 0.90)	0.90 (0.88; 0.93)	0.92 (0.86; 0.99)
	5	0.81 (0.77; 0.84)	0.80 (0.77; 0.83)	0.87 (0.81; 0.93)
	6	0.78 (0.74; 0.82)	0.80 (0.78; 0.83)	0.84 (0.79; 0.90)
	7	0.79 (0.75; 0.82)	0.77 (0.75; 0.80)	0.86 (0.80; 0.92)
	8	0.78 (0.75; 0.82)	0.78 (0.75; 0.81)	0.82 (0.76; 0.88)
	9	0.71 (0.67; 0.74)	0.70 (0.67; 0.72)	0.77 (0.72; 0.82)
	10	0.57 (0.54; 0.59)	0.61 (0.59; 0.64)	0.72 (0.68; 0.78)
**PLA TO THE GYNAECOLOGIST** (TERCILES)	1[1]	1	1	1
	2	0.89 (0.87; 0.91)	1.11 (1.09; 1.13)	1.12 (1.10; 1.15)
	3	0.76 (0.75; 0.78)	0.96 (0.94; 0.98)	1.25 (1.22; 1.28)

[1]*: Reference Category*

Table 5: **GP consultation during the year among women with and without a RP in Midi Pyrénées** (2012)

In population A: women over 16 years old (n= 1,072,289)

	No RP<br>n =98258<br>N(%)	RP<br>n = 974031<br>N(%)	OR = 10.18<br>95% CI [10.04; 10.33]<br>p-value < 0.001
**No GP consultation**	65165 (66.32)	157809 (16.20)	
**≥ 1 GP consultation**	33093 (33.70)	816222 (83.80)	

Table 6: **Mammography uptake and GP visit during the year among women aged 50-74 with or without RP in Midi Pyrénées** (2012)

In population B: 50-74 (n=365,947)

Women who had ≥ 1 mammography within the year	**No GP consultation**<br>N(%)	**≥1 GP consultation**<br>N(%)	
**Total**<br>112,593 (30.77%)	9219 (14.80)	103374 (34.04)	OR = 2.97 95%CI [2.90; 3.04]<br>p-value < 0.001
**No RP**	281 (1.80)	788 (17.72)	
**RP**	8938 (19.15)	102586 (34.28)	

Table 7: **Pap smear and GP visit in the past year among women aged 25-65 with or without RP in Midi Pyrénées** (2012)

In population C: 25-65 women (n=711,803)

Women who had ≥ 1 pap smear within the year	**No GP consultation**<br>N(%)	**≥ 1 GP consultation**<br>N(%)	
**Total**<br>205,072 (28.81%)	23847 (15.45)	181225 (32.51)	OR = 2.64 95%CI [2.60; 2.67]<br>p-value < 0.001
**No RP**	1558 (3.76)	3090 (19.13)	
**RP**	22289 (19.75)	178135 (32.91)	
